# Detection Range of Acoustic Monitoring Devices in an Open Grassland Ecosystem

**DOI:** 10.1002/ece3.74181

**Published:** 2026-08-15

**Authors:** Grace E. Schuster, Leroy J. Walston, Andrew R. Little

**Affiliations:** ^1^ School of Natural Resources University of Nebraska‐Lincoln Lincoln Nebraska USA; ^2^ Environmental Science Division Argonne National Laboratory Lemont Illinois USA

**Keywords:** acoustic recording units (ARUs), detection space, grasslands, passive acoustic monitoring (PAM)

## Abstract

Passive Acoustic Monitoring (PAM) is a novel method for estimating avian biodiversity, producing results comparable to traditional methods, such as point count surveys. However, uncertainty in the detection space around acoustic recording units (ARUs) can complicate monitoring designs and potentially lead to biased results. Therefore, understanding ARU detection space in different environments is essential to advancing the reliability of PAM for avian population monitoring. In this study, we used a distance‐based broadcasting method at seven distances from ARUs in grassland systems in eastern Nebraska, USA, to quantify the detection space for six focal species common in this landscape. We found that detection space was species‐specific but, overall, most confirmed detections (detection probability ≥ 0.50) occurred within 100 m (m) of the ARU. Detectability was positively influenced by amplitude, while the effect of vocalization frequency varied, producing species‐specific differences in detectability. Detection space is a highly variable yet essential consideration in the design of large‐scale PAM systems, and its estimation should be a key first step in any PAM study. We demonstrate a replicable method for assessing detection space that can be applied in other ecosystems and for other bird species. Although more labor‐intensive than other approaches, this method provides reliable estimates of detection space, which are important considerations for the ongoing application of PAM technologies for biodiversity conservation.

## Graphical abstract

Understanding acoustic recording units (ARUs) detection space in different environments is essential to advancing the reliability of passive acoustic monitoring (PAM) for avian population monitoring. In this study, we used a distance‐based broadcasting method at seven distances from ARUs in grassland systems to calculate the optimal detection distance for six focal species common in this landscape. We found that detection space was species‐specific, but should be considered in any large‐scale PAM system.

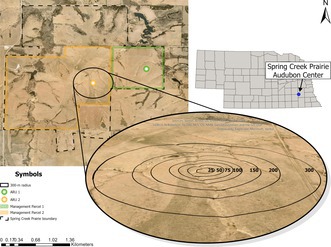

## 1 Introduction

The 21st century has been marked by rapid technological advancement alongside accelerating environmental change. While innovations in artificial intelligence (AI) and computing power have transformed nearly every sector of society, this period has also witnessed an unprecedented decline in global biodiversity (Hooper et al. 2012). Human pressures on natural systems have intensified, making the need for timely, accurate, and large‐scale biodiversity monitoring more critical than ever. Responding to this pressing need, passive acoustic monitoring (PAM) has emerged as a promising approach to generating timely and reliable biodiversity estimates (Sugai et al. 2019; Teixeira et al. 2024). In addition, advances in machine learning methods for identifying vocalizations of interest (Kahl et al. 2021), have led many ecologists to embrace PAM as a promising alternative to traditional point count surveys (Shonfield and Bayne 2017). PAM methods can produce avian population estimates (i.e., species richness) similar to point count surveys (Schuster et al. 2024) while reducing some of the observer biases and logistical challenges related to traditional field surveys (Sugai et al. 2019). As the application of this emerging technology continues to spread, it is important that we expand our understanding of monitoring system capabilities in different environments.

Biodiversity inferences generated from PAM are dependent upon the context of the detection space sampled by acoustic recording units (ARUs; Darras et al. 2016). “Detection space” is defined as the distance sampled by an acoustic monitoring system (Llusia et al. 2011). This area can vary based on call characteristics of individual bird species (Rempel et al. 2013) as well as vegetation height and density (Darras et al. 2016). Biodiversity estimates (e.g., species richness, diversity, etc.) depend on methods that operate at specific spatial scales. Understanding the spatial area sampled by ARUs is crucial for interpreting these estimates; failure to do so can lead to biased results (Van Wilgenburg et al. 2017). For example, assuming that detection space is identical across vegetation types and species can lead to either overestimation or underestimation of true detection probabilities, ultimately introducing bias into occupancy estimates (MacKenzie et al. 2002). Assessing detection space is also crucial for PAM study design, as it informs the effective distance between ARUs and how close they can be positioned to habitat edges (Hingston et al. 2018). Recent ornithological studies employing PAM have placed ARUs anywhere from 100 to 500 m (m) apart (Darras, Batáry, et al. 2018; Darras, Furnas, et al. 2018; Dixon et al. 2023; Ware et al. 2023; Schuster et al. 2024; Walston et al. 2025). Most of these studies assume a detection range based on previous research from other applications rather than site‐specific in situ testing of ARUs (but see Furnas and Callas 2015). Given the potential influence of the surrounding environment (e.g., vegetation height and density) on ARU detection space and the wide variation of assumed detection spaces, biodiversity acoustic surveys would benefit from a standardized, replicable approach for assessing detection spaces across multiple ecosystems.

Several studies have attempted to estimate the detection space of ARUs across different species and landscape types. Detection space between point counts and ARUs have been compared in previous studies to estimate and characterize differences in detection space (Hutto and Stutzman 2009; Hingston et al. 2018; Yip et al. 2017; Darras, Batáry, et al. 2018), and in some cases integrated to account for method‐specific detection biases (Van Wilgenburg et al. 2017). In a boreal forest in Saskatchewan Canada, Van Wilgenburg et al. (2017) found that ARUs are capable of detecting bird vocalizations at distances < 69‐m. Several other studies in forest systems have reported an ARU effective detection distance of approximately 50‐m (Venier et al. 2012; Furnas and Callas 2015; Van Wilgenburg et al. 2017), with some suggesting that the majority (95%) of bird calls are detectable only within 40‐m (Darras, Furnas, et al. 2018). Previous studies have shown that species‐specific traits can influence detection space. Winiarska et al. (2024) estimated detection probabilities for 31 forest bird species and found that detectability declined with distance while also varying according to species‐specific traits, including body mass and vocal characteristics such as call frequency. In Indonesian forests, Darras et al. (2016) noted that the source level of vocalizing species and the diversity of their repertoires will also influence detection space. Although ARU detection space has been studied previously, primarily in forested environments, there remains a knowledge gap regarding ARU detection space in open grassland systems. Probability of detection with ARUs has been shown to decline more rapidly in dense vegetation than in more open habitats (Yip et al. 2017). As a result, detection space may be greater in grassland ecosystems, an environment where empirically based detection data is limited.

In this study, we aimed to demonstrate a replicable approach for estimating the detection space of ARUs in grassland systems for several avian species, although the method is broadly applicable to other environments. Due to our interest in estimating detection spaces for specific species, we used distance‐based broadcasting, which involves playing vocalization clips at known distances and amplitudes from the recorder (Yip et al. 2017). Although this method can be more labor‐intensive than alternative approaches, it has the advantage of using calls at known distances rather than relying on observer‐estimated distances, which may be subject to uncertainty (Alldredge et al. 2007). It is also a more feasible method than ARU‐human point count comparisons for estimating detection space for species that are rare or less vocal and therefore have fewer detections. The objectives of our study were to: (1) demonstrate a reproducible methodology for estimating ARU detection space to inform PAM study design; (2) quantify the detection space of ARUs for six focal species within an open grassland ecosystem; and (3) determine optimal distances to achieve desired detection probabilities for focal species of interest. We define “optimal distance” as the minimum distance between the ARU and the vocalization source required to achieve a specified detection probability. We predicted that (a) detection spaces could be quantified for our focal species and (b) these detection spaces in open grassland systems in our study would be larger than previously estimated detection spaces in closed forested environments (i.e., Venier et al. 2012; Hingston et al. 2018; Winiarska et al. 2024).

## 2 Methods

### 2.1 Study Area

**FIGURE 1:**
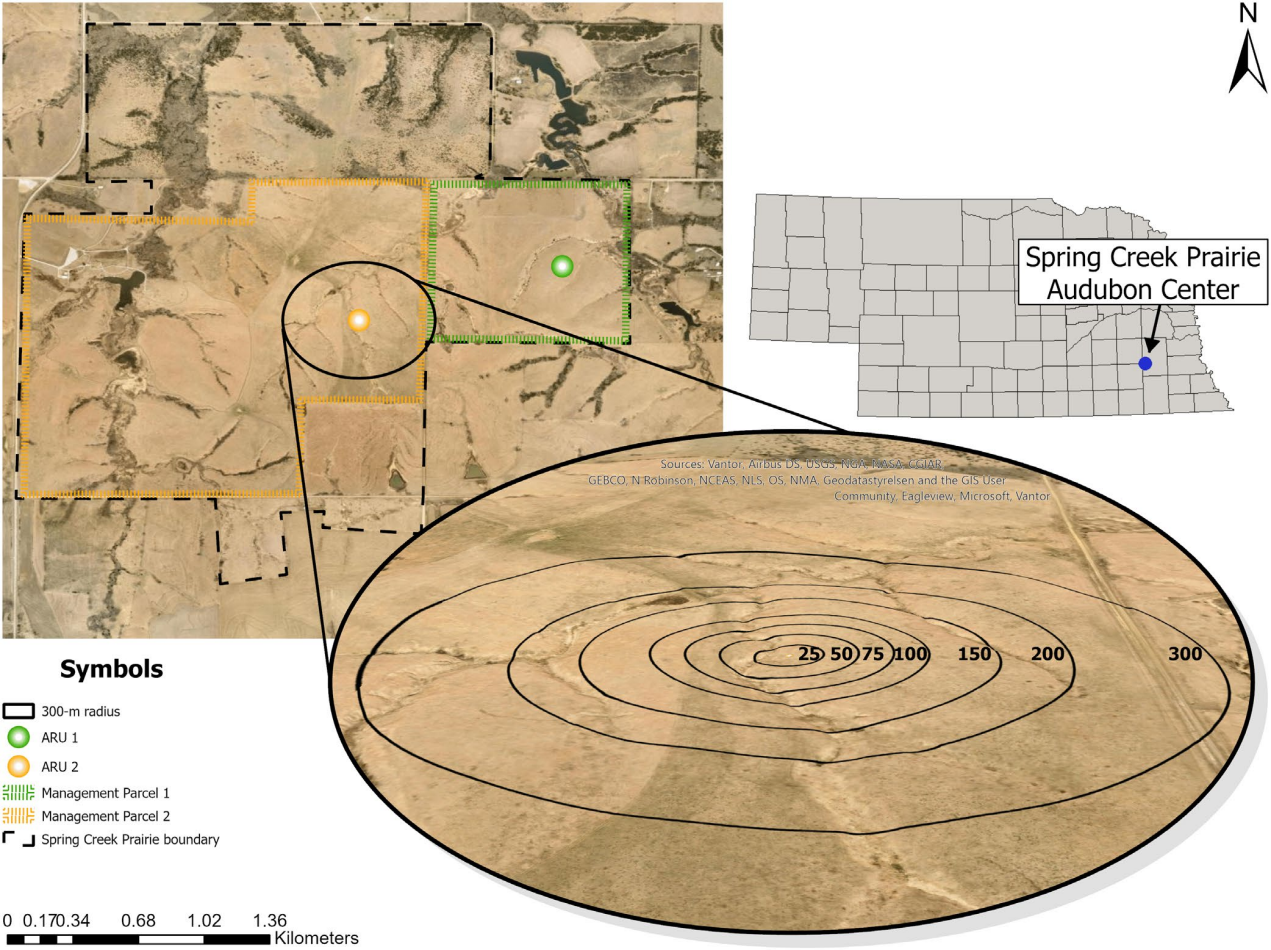
A map of the seven distances tested around the ARUs in two different parcels at Spring Creek Prairie Audubon Center in eastern Nebraska.

The detection distance experiment was conducted at Spring Creek Prairie Audubon Center in Denton, Nebraska, USA, a 1160‐acre (469.43‐ha) public property managed as native tallgrass prairie. Between October 1 and October 13, 2025, we conducted field experiments to examine ARU detection space in two parcels (Figure 1). The dominant vegetation in both parcels consisted of Big Bluestem ( *Andropogon gerardii* ) and Smooth Brome ( *Bromus inermis* ), and average vegetation height in both parcels was less than 1 m. The parcels were managed differently over the past 2 years. In both 2024 and 2025, one parcel was partially harvested for hay production, while the other was grazed in both years.

### 2.2 Field Methods

Two ARUs (Wildlife Acoustic Song Meter Minis, Maynard, MA, USA; Figures S1 and S2) were installed at random locations within the two grassland parcels, each positioned ≥ 100‐m from the field edge (Figure 1). The ARUs were > 1000‐m from one another. One ARU was an earlier generation Song Meter Mini (hereafter, ARU #1), while the other was the updated model, the Song Meter Mini 2 (hereafter, ARU #2). Despite these model differences, there were no reported hardware or programming differences between these two recorders. The only differences were cosmetic (enclosure design) and battery arrangement (Song Meter Mini 2 User Guide 2024). Both ARUs were programmed to collect 16‐bit WAV audio at a sample rate of 24,000 Hz, continuously, with a gain setting of 18 dB (dB), however; the units were not calibrated. The monitors were attached to a polyvinyl chloride (PVC) pole placed over a steel metal fence post, approximately 1.5‐m above the ground and both ARUs faced North.

We selected three different prerecorded 5‐s vocalization acoustic clips for six focal species: Dickcissel ( *Spiza americana* ; hereafter “SPAM”), Grasshopper Sparrow ( *Ammodramus savannarum* ; hereafter “AMSA”), Northern Bobwhite ( *Colinus virginianus* ; hereafter “COVI”), Ring‐necked Pheasant ( *Phasianus colchicus* ; hereafter “PHCO”), Wild Turkey ( *Meleagris gallopavo* ; hereafter “MEGA”), and Mourning Dove ( *Zenaida macroura* ; hereafter “ZEMA”). These were assembled into three 30‐s clips, each containing a sequence of 5‐s vocalization segments from the six focal species, with the order of species varying among clips. These focal species are common grassland and habitat generalist species in Nebraska. Vocalizations were selected from previously collected acoustic recordings from southwestern Nebraska (Schuster et al. 2024, 2025). To broadcast test acoustic clips, we used a FoxPro Shockwave speaker system (Lewistown, PA, USA; https://www.gofoxpro.com/).

To assess detection distance for the six focal species, we conducted playbacks in four cardinal directions (S, E, W, N) around the ARU. In each direction, the three 30‐s clips were broadcast at seven distances (25‐, 50‐, 75‐, 100‐, 150‐, 200‐, & 300‐m; Yip et al. 2017). Each clip was played at three volume levels: low, medium, and high, resulting in nine 30‐s playbacks per distance in each cardinal direction. We configured our playback volume settings (re: 20 𝜇Pa) to be inclusive of source levels considered to be produced by vocalizing focal species. To assess the dB ranges for each playback level (low, medium, high), we measured the maximum dB of the three 5‐s clips for each species using a sound level meter (XC12 Sound Level Meter, Guangdong Province, China), held at 1‐m away from the speaker, and calculated the mean maximum dB across the three trials. Across all species and playback calls, low playback levels ranged from 51.27–82.20 dB (mean = 62.80, Coefficient of Variation (CV) = 0.14), medium playback levels ranged from 60.23–107.10 dB (mean = 80.48, CV = 0.14), and high playback levels ranged from 74.93–128 dB (mean 104.26, CV = 0.15; Table S1). Although the volume at which wild birds sing is relatively unknown (Brackenbury 1979), the mean maximum amplitude of our “medium” group was consistent with playback levels used in previous studies assessing detection space (Yip et al. 2017; Shaw et al. 2022). The “high” and “low” groups were then defined relative to this medium baseline, providing a structured range of playback amplitudes for comparison.

The FoxPro speaker was held approximately 1‐m above the ground and oriented toward the ARU during all playbacks in the field. Playbacks were initiated at the top of a minute, and the playback time was recorded so it could be quickly identified within the corresponding audio file. Any naturally occurring focal species vocalizations during the playback experiment were documented to avoid potential misidentification. Wind speed was measured with a Kestrel (Kestrel 3000 Weather Meter, Boothwyn, PA, USA; KestrelMeters.com) prior to each playback. We completed two full rounds of distance measurements in each cardinal direction at each ARU, starting with the closest distance (25‐m) during the first round and the farthest distance (300‐m) during the second round.

### 2.3 Manual Annotation of Recordings

We manually annotated all recordings from each round of detection experiments. One observer listened to each recording and noted whether the focal species' vocalizations were present or absent. If the focal species' vocalization was clearly audible, meaning it was distinct from background noise, and could be confidently identified based on its characteristic acoustic features (e.g., frequency, patterns) in the recording, it was documented as present at a specific distance and playback level. If the vocalization could not be clearly detected or identified, it was recorded as absent in the recording.

### 2.4 Statistical Methods

We assessed detection probability using Generalized Linear Mixed Models (GLMMs) to predict presence or absence of playback vocalizations across all seven distances and three playback levels for each ARU and five of the six focal species. We did not include the low playback level for ZEMA due to lack of detections. Models were fitted with a binomial error distribution and a logit link function. Wind speed was incorporated as a random effect (*R*_*j*_); based on its distribution (mean = 4.7 ± 2.9 km/h; range = 0–19.8), we categorized wind speed measurements into low, medium, and high levels. Wind speed was included as a random effect because it is an uncontrolled environmental factor. Therefore, we simplified it into categories and modeled it as random variability rather than a precise, interpretable predictor. We developed four models to understand variability in focal species vocalization presence in the recordings, along with a global and control model (Table 1). Model structure was informed by a priori assessment of plausible interactions, as well as exploratory model comparisons. Because the distance at which a vocalization could be detected was expected to depend on both ARU and playback level, we included interaction terms among these variables. We then compared models with and without interactions and found that those including interactions among playback level, ARU, and distance consistently outperformed models with only independent effects. Distance was also retained as an independent term in all models due to its central importance to the study objectives. The “glmer” function from the “lme4” R package (Bates et al. 2015) was used to generate GLMMs.

**TABLE 1:** Candidate models used to predict presence or absence of vocalizations across seven distances, three playback levels, and two ARUs for the six focal species. *ZEMA* was the only species tested at only two playback levels.

Model No.	Equations
1	Presence ~ Distance + *Rj*
2	Presence ~ Distance * Playback Level + *Rj*
3	Presence ~ Distance * ARU + *Rj*
4	Presence ~ Distance * ARU + Distance * Playback Level + *Rj*
5	Presence ~1 + *Rj*
6	Presence ~ Distance * ARU + Distance * Playback Level + ARU * Playback Level + *Rj*

*Note:* *Rj* denotes random variables included in the model.

Models were compared using second‐order Akaike Information Criteria (AICc), which accounts for small sample size, to identify the best‐supported model for probability of detection of each focal species (Anderson and Burnham 2002). We considered models to have substantial support when their ΔAICc was less than 2.0 units away from the top model, and we calculated adjusted Akaike weights (*w*_i_) for all competitive models. Top‐performing models were model averaged using the “MuMIn” package (Bartoń 2010) and then were used to make model predictions. Based on these predictions, we estimated optimal distances as the minimum distance at which each focal species reached target detection probability thresholds of 0.25, 0.50, and 0.75.

We compared detection probabilities between the two ARUs since they were installed in different parcels with different vegetation structures and represented different hardware versions. The “emmeans” package in R (Lenth and Piaskowski 2025) was used to estimate and compare Estimated Marginal Means (EMMs), generating predicted detection probabilities for each combination of playback level and ARU for all six focal species. This package was also used to make pairwise comparisons of detection probabilities for each focal species in each tested direction (S, E, W, N) around the ARU. We evaluated cardinal direction as a nuisance variable to account for potential microphone placement biases. To visualize the frequency of focal species vocalizations, spectrograms were created using the “av” package (Ooms 2025). All analysis was completed in R version 4.5.1 (R Core Team 2021).

## 3 Results

**FIGURE 2:**
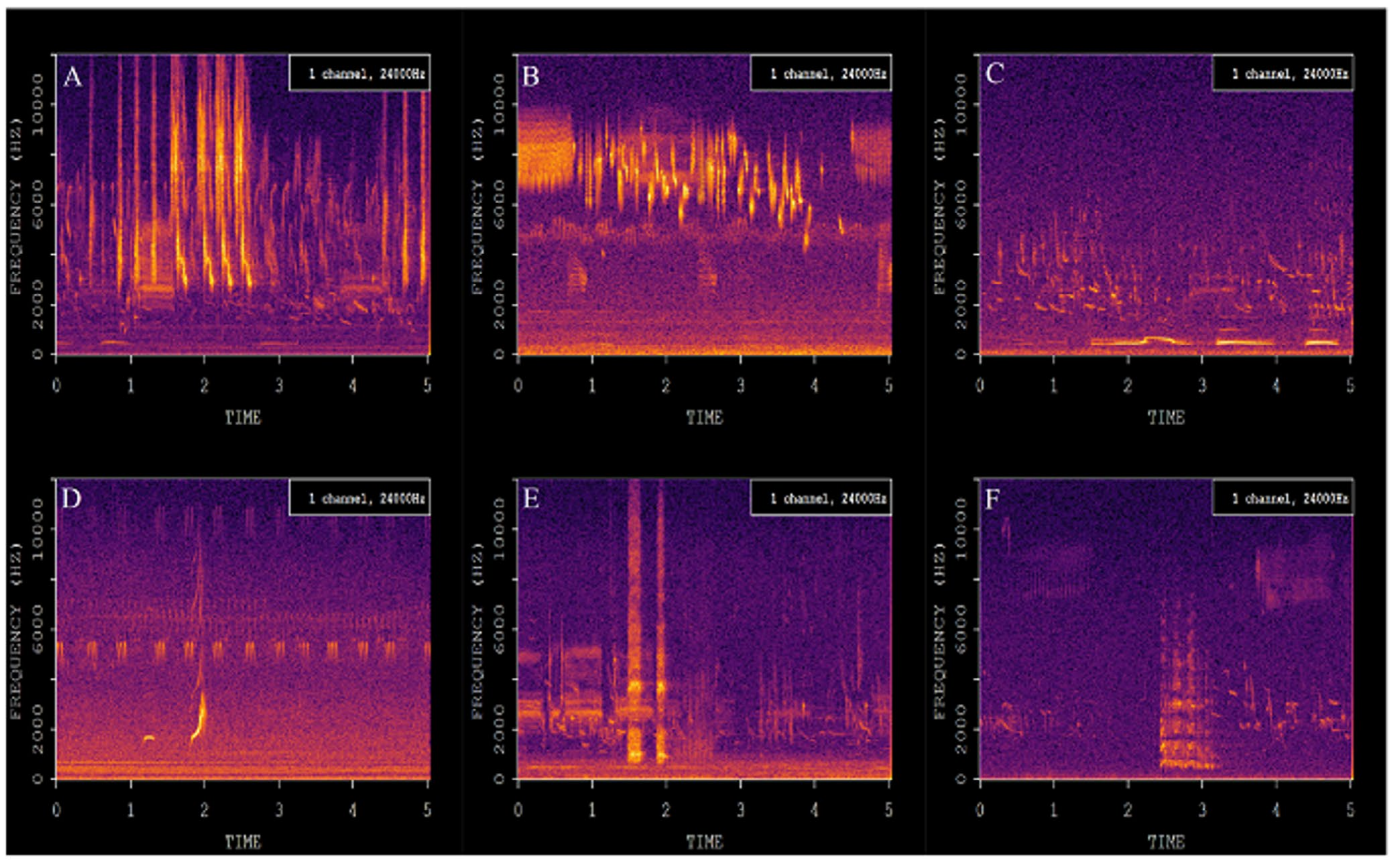
Spectrograms of vocalizations used in the playback experiment for the six focal species. Panels depict the following species: (A) Dickcissel (SPAM), (B) Grasshopper Sparrow (AMSA), (C) Mourning Dove (ZEMA), (D) Northern Bobwhite (COVI), (E) Ring‐necked Pheasant (PHCO), and (F) Wild Turkey (MEGA). Warmer colors (e.g., orange and pink) represent higher amplitude sounds (dB), while cooler colors indicate lower amplitude sounds.

Detection range experiments were conducted over 5 days, during which we collected approximately 34 h of acoustic data containing playbacks. Of the 6048 possible playback detections, 70.2% were not manually confirmed on the recordings because they were not clearly detectable in the recordings, and 29.7% were confirmed. Six detections were misplayed and therefore removed from the data set. Low wind speed ranged from 0.0 to 3.0 km/h, medium wind speeds from 3.2 to 5.3 km/h, and high wind speed from 5.5 to 19.8 km/h. The vocalization frequencies of focal species ranged from ≤ 2000 Hz to ≥ 10,000 Hz (Figure 2).

**FIGURE 3:**
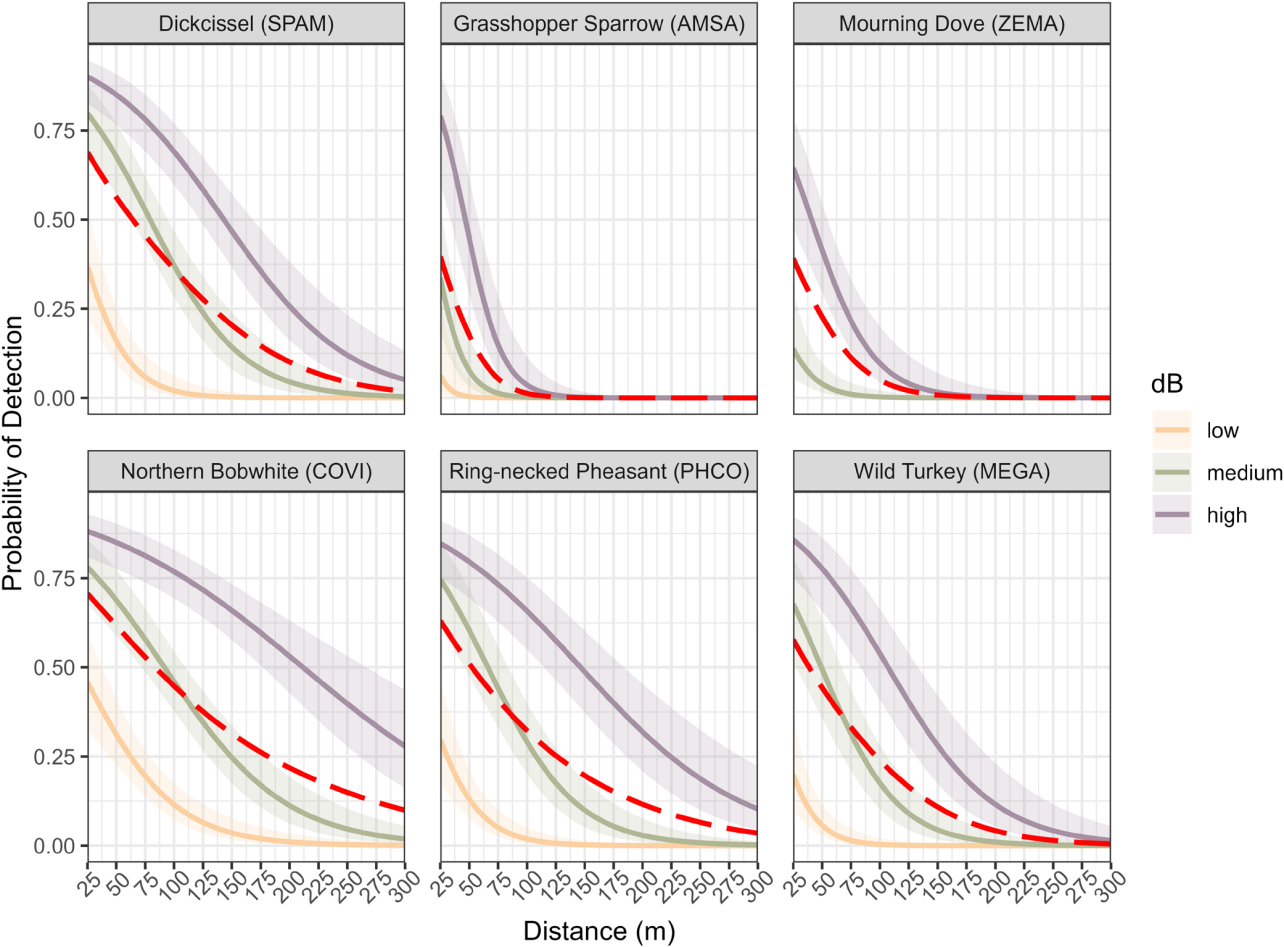
Predictions based on the top‐performing model(s) for each focal species across playback levels. The red dashed line represents average detection probability across playback levels for each species. “Distance” represents the distance between the monitor and the source of the vocalization.

The top‐performing model for PHCO, COVI, and MEGA was ~ Distance * ARU + Distance * Playback Level + Rj (Tables S2–S4). All variables in the PHCO and COVI models were significant (*p* ≤ 0.001). In the MEGA model, only distance, playback level, and the interaction between distance and ARU were significant (*p* ≤ 0.001). The top‐performing model for SPAM, AMSA, and ZEMA was also ~ Distance * ARU + Distance * Playback Level + Rj followed closely by the Global Model (Tables S5–S7). Distance was a significant predictor for all focal species (*p* ≤ 0.001) and as distance increased, detection probability decreased across all playback levels and for both ARUs (Figure 3).

For PHCO and COVI, ARU #1 exhibited substantially lower overall detection probability than ARU #2 (44% and 52% lower odds, respectively); however, detection probability at ARU #1 declined more slowly with increasing distance (*p* < 0.01; Figure S3). Furthermore, for MEGA and SPAM, overall detection probability did not differ significantly between ARUs, but detection at ARU #1 was less negatively affected by distance. In contrast, AMSA had 92% lower odds of detection at ARU #1 compared to ARU #2 (*p* < 0.01), with no evidence that the effect of distance differed between ARUs. Detection probability of ZEMA did not differ between ARUs, and the effect of distance on detection was consistent across ARUs.

The high playback level had the greatest percent probability of detection for all focal species, followed by medium and low, although this relationship was not significant in all top‐performing models (Tables S2–S7). PHCO and COVI were the only species detected at 300‐m, and each was detected only during high playback trials. For all focal species, wind speed had minimal effect on detection probability.

Based on top‐performing model predictions, optimal distances for target probabilities were estimated (Table 2). For a 25% target probability, the average optimal distances across species are 34.1–, 86.8‐, and 170.0–m at low, medium, and high playback levels, respectively. At a 50% target probability, the corresponding distances decrease to 25.0–, 56.0–, and 116.0–m. For a 75% target probability, they decline further to 25.0–, 28.3–, and 62.0–m across the same playback levels.

**TABLE 2:** The optimal detection distances for detection probabilities of 25%, 50%, and 75% for all focal species generated from the top‐performing models.

Species name	Species code	Playback level	Minimum predictive P_d_	Maximum predictive P_d_	Optimal distance (P_d_ = 0.25)	Optimal distance (P_d_ = 0.50)	Optimal distance (P_d_ = 0.75)
Ring‐necked Pheasant	PHCO	Low	0.00	0.30	30.81	< 25.00	< 25.00
		Medium	0.00	0.75	107.93	66.09	< 25.00
		High	0.10	0.85	224.16	146.36	68.56
Northern Bobwhite	COVI	Low	0.00	0.46	62.40	< 25.00	< 25.00
		Medium	0.02	0.78	149.34	91.58	39.57
		High	0.28	0.88	306.87	211.55	109.30
Wild Turkey	MEGA	Low	0.00	0.20	25.00	< 25.00	< 25.00
		Medium	0.00	0.68	85.33	49.31	< 25.00
		High	0.01	0.86	157.25	107.06	56.86
Grasshopper Sparrow	AMSA	Low	0.00	0.09	25.00	< 25.00	< 25.00
		Medium	0.00	0.35	31.85	< 25.00	< 25.00
		High	0.00	0.76	63.48	44.76	26.03
Mourning Dove	ZEMA	Low	0.00	0.01	< 25.00	< 25.00	< 25.00
		Medium	0.00	0.16	< 25.00	< 25.00	< 25.00
		High	0.00	0.65	69.85	41.09	< 25.00
Dickcissel	SPAM	Low	0.00	0.36	36.33	< 25.00	< 25.00
		Medium	0.00	0.80	121.58	78.89	36.20
		High	0.05	0.90	202.74	144.59	86.43

*Note:* Optimal distance is in meters.

Abbreviation: P_d_, Probability of detection.

Based on the EMMs, ARU #2 had a higher percent probability of detection for all focal species, although the difference was not always significant (Table S8). For PHCO, MEGA, and COVI detection probability did not differ significantly among ARUs at medium playback levels. SPAM had higher detection probability at ARU #2 at all playback levels, whereas AMSA did not differ significantly between ARUs at any playback level. ZEMA had higher detection probabilities at ARU #2 at medium and high playback levels. Although higher playback levels increase detection probabilities for both ARUs, this does not account for variations over distance.

There was considerable variation in predicted detection probabilities among ARU directions across species (Tables S9 and S10), although only 9 out of 36 pairwise comparisons were significantly different from one another. Overall, the southern direction generally had the highest detection probability; although differences were not always statistically significant.

## 4 Discussion

Previous efforts to quantify detection space of ARUs have mostly been conducted in forested or densely vegetated environments, leaving detection space in open grassland habitats largely unknown. Common approaches have involved integrating human‐derived point counts with simultaneous ARU sampling to estimate the distance at which bird vocalizations can be detected. However, these methods rely on the assumption that observer‐estimated distance to calling birds is accurate (Alldredge et al. 2007), which can introduce substantial uncertainty. We demonstrate a more standardized and replicable approach for estimating ARU detection space that can be applied across various ecosystems and avian species. By broadcasting vocalizations of six focal species at known distances from ARUs, we provide species‐specific and probability‐based optimal detection distances. This playback method offers a transferable approach for characterizing detection space and can be implemented across study areas to improve study design and comparability of population estimates.

Detection distances for the focal species in this study varied considerably based on playback level and desired probability of detection. The optimal distances associated with 50% detection probability for all six species were < 100‐m at medium playback levels (Table 2). This finding can assist researchers in the design of future PAM studies by providing an example benchmark to guide the placement of ARUs. For target species, grassland systems, and ARUs similar to those we investigated in this study, installing ARUs 100‐m apart or away from one another or from landcover edges could help achieve statistical independence in recorded species. However, optimal spacing may vary depending on several site‐specific biological and environmental factors. For example, for studies focusing on relatively quiet‐vocalizing species (e.g., AMSA), the effective detection space may be smaller than 100‐m. The medium playback levels we used (mean = 80.48 dB) were similar to volumes used in previous studies assessing detection space (Yip et al. 2017; Shaw et al. 2022). Our results are consistent with some previous studies but contrast with others conducted in forested environments. For example, Hingston et al. (2018) found that the detection space of ARUs in Tasmanian forested systems was 100‐m. In contrast, Venier et al. (2012) reported detection rates of 59% for individuals within 25‐m and 39% for those at distances of 51–100‐m, but did not include any distances greater than 100‐m. Winiarska et al. (2024) also used a playback‐based approach rather than live birds and found that a 50% detection probability was achieved in coniferous and deciduous forests at distances of approximately 150–200‐m. This suggests that vegetation structure and/or land use may not be the only influential predictors of ARU detection space relative to other factors, such as species–specific call amplitude and frequency, and therefore sound transmission should be measured directly in every unique environment (Darras et al. 2016). Although overall detection space of ARUs was similar between forest and grassland habitats, detection probability may decline less rapidly in open vegetation (Yip et al. 2017).

It is not surprising that as distance from ARU increased, overall detection probability decreased (Darras, Furnas, et al. 2018; Winiarska et al. 2024; Figure 3); but with greater sound pressure levels, probabilities decreased more gradually. Consequently, species that vocalize at higher amplitude levels are more likely to be detected at farther distances from the ARU than quieter species (Winiarska et al. 2024). This detection bias must be considered when interpreting ARU results and their ecological implications. Wind speed had little effect on detection in our analysis. Similarly, Yip et al. (2017) found that wind speed was not included in the top‐performing models for estimating detection probability across 23 boreal bird species, suggesting it is less influential than distance from the vocalization source. Detectability may not depend solely on source level but also on vocalization frequency (Gibb et al. 2019) and environmental noise. ZEMA playback calls consisted of primarily low frequencies (< 2000 Hz; Figure 2). Previous studies indicate that detectability decreases with increasing frequency (Yip et al. 2017; Winiarska et al. 2024); however, ZEMA exhibited a smaller detection radius compared to the other focal species. Therefore, it is possible that environmental noise masked ZEMA calls, particularly given that masking effects are most pronounced in lower‐frequency ranges (Fossesca et al. 2023). This could have reduced detectability and contributed to the smaller estimated detection radius for this species.

There was also a difference in detection between ARUs, which could be due to a few reasons that were not a part of the variables tested here. ARU #1 did have lower detection probability for some of the species (PHCO, COVI, and AMSA), but the effect of distance was not as strong. One possible explanation could be due to degraded microphone sensitivity because ARU #1 was an older model that has been previously used in the field, whereas ARU #2 was a newer model (Turgeon et al. 2017). Vegetation structure may also help explain the observed differences. ARU #1 was located in a parcel that was only partially hayed over the past 2 years, whereas ARU #2 was placed in a parcel that was fully grazed during the same period. The shorter vegetation likely present in the grazed parcel may have improved detection probability for certain species. However, because vegetation data were not collected and included in the model, this relationship cannot be confirmed. Detection probabilities varied among directions and were highest in the southern direction. Previous research has noted that microphones are biased toward the direction they face (Shaw et al. 2022) in this case the microphones were facing west, suggesting that other factors influenced detection. We found limited evidence that ARU directionality is an important variable influencing detection space. However, since we only tested two ARUs these conjectures cannot be confirmed. Our study had a limited sample size and did not account for other factors, such as wind direction; therefore, the relationships described here may vary if these metrics were considered.

While our approach is labor intensive, it has the benefits of being replicable, does not assume distance of vocalization, and can be used to test detection space for less vocal species. There are some shortcomings to our methods that should be discussed. PHCO and COVI were both confirmed at high playback levels at the maximum tested distance from the ARU. Therefore, it may be beneficial to expand the tested distance to > 300‐m in open systems, for example, Yip et al. (2017) tested distances up to 800‐m. Furthermore, low playback ZEMA calls were not confirmed at any distance, signifying that the low‐level volume we chose may have been too low. Playing back vocalizations at > 60 dB for the lowest setting may be better for future studies. We relied on previous literature (Yip et al. 2017; Shaw et al. 2022) to estimate source levels and establish a representative medium playback volume for each species. This approach may have misrepresented the natural variation in call amplitude expressed by birds in the wild. Future studies should consider conducting in situ measurements of vocalizations from focal species or standardizing playback levels to minimize variability and improve relevance. Finally, researchers employing these methods should be cognizant of the large amount of acoustic data that can be generated from this approach and need to consider the impacts of ARU detection space on data storage and analytical workflows.

## 5 Conclusion

The detection space of an ARU is influenced by a range of factors; however, if it is unaccounted for, this variation can result in biased biodiversity estimates (Gibb et al. 2019). This study provided optimal distances to obtain target detection probabilities in grassland ecosystems for the six focal species of interest. If the species of interest is included in this study, we recommend using Table 2 to estimate the placement of ARUs and account for differences in detectability. We also demonstrated a method for effectively estimating detection space that minimizes observer bias and can be applied in various environments. No single ARU distance can ensure perfect detection due to substantial variation in avian bioacoustics and spatiotemporal factors. The most effective way to increase detection space is to expand recording schedules over broader temporal scales, providing more opportunities for detection. However, considering detection space should be a necessary first step in any large‐scale PAM scheme.

## Author Contributions

**Grace E. Schuster:** conceptualization (equal), formal analysis (equal), investigation (equal), methodology (equal), validation (equal), visualization (equal), writing – original draft (equal), writing – review and editing (equal). **Leroy J. Walston:** conceptualization (equal), formal analysis (equal), methodology (equal), resources (equal), writing – original draft (equal), writing – review and editing (equal). **Andrew R. Little:** funding acquisition (equal), project administration (equal), resources (equal), supervision (equal), writing – review and editing (equal).

## Funding

This work was supported by Bioenergy Technologies Office (DE‐EE0009279).

## Conflicts of Interest

The authors declare no conflicts of interest.

## Supporting information


**Data S1:** Supporting Information.

**Table S1:** The average maximum dB of each focal species clip played during the experiment. On the FoxPro, the volume settings were adjusted as follows: 14 for low playback volume, 27 for medium playback volume, and 40 for high playback volume, which is the maximum volume setting on the device.
**Table S2:** For Ring‐necked Pheasant, Model 4 was the top‐performing model according to the full model output. Asterisks denote significance of variables in the model (* ≤ 0.05, ** ≤ 0.01, *** ≤ 0.001) and playback level is represented by dB.
**Table S3:** For Northern Bobwhite, Model 4 was the top‐performing model according to the full model output. Asterisks denote significance of variables in the model (* ≤ 0.05, ** ≤ 0.01, *** ≤ 0.001) and playback level is represented by dB.
**Table S4:** For Wild Turkey, Model 4 was the top‐performing model according to the full model output. Asterisks denote significance of variables in the model (* ≤ 0.05, ** ≤ 0.01, *** ≤ 0.001) and playback level is represented by dB.
**Table S5:** For Dickcissel, Models 4 and 6 were the top‐performing models according to the full mode output. Asterisks denote significance of variables in the model (* ≤ 0.05, ** ≤ 0.01, *** ≤ 0.001) and playback level is represented by dB.
**Table S6:** For Grasshopper Sparrow, Models 4 and 6 were the top performing models according to the full model output. Asterisks denote significance of variables in the model (* ≤ 0.05, ** ≤ 0.01, *** ≤ 0.001) and playback level is represented by dB.
**Table S7:** For Mourning Dove, Models 4 and 6 were the top‐performing models according to the full model output. Asterisks denote significance of variables in the model (* ≤ 0.05, ** ≤ 0.01, *** ≤ 0.001) and playback level is represented by dB. Low dB was not included in these models.
**Table S8:** Detection probability for each ARU and focal species across playback levels. Asterisks denote significance between ARUs (* ≤ 0.05, ** ≤ 0.01, *** ≤ 0.001).
**Table S9:** Probability of detection for each direction and focal species.
**Table S10:** Significant differences in detection probability between directions for each focal species. Asterisks denote significance between ARUs (* ≤ 0.05, ** ≤ 0.01, *** ≤ 0.001).
**Figure S1:** ARU #1 in Management Parcel #1.
**Figure S2:** ARU #2 in Management Parcel #2.
**Figure S3:** Predictions based on the top‐performing model(s) for each focal species across ARUs, without incorporating variation in playback level. The red dashed line represents average detection probability across ARU for each species. “Distance” represents the distance between the monitor and the source of the vocalization.

## Data Availability

All the required data are uploaded as Supporting Information.
