# Supplementary material for: Detection Range of Acoustic Monitoring Devices in an Open Grassland Ecosystem
Source: Ecol Evol. 2026 Aug 15;16(8):e74181. doi: 10.1002/ece3.74181 (PMC13476758; doi:10.1002/ece3.74181)
Supplement: Supplementary file 1 [file ECE3-16-e74181-s002.docx]

**SUPPLEMENTAL INFORMATION**

Detection Range of Acoustic Monitoring Devices in an Open Grassland Ecosystem

**Supplemental Material**

Table of Contents___________________________________________________________________SI-2

Table S1__________________________________________________________________________SI-3

Table S2__________________________________________________________________________SI-3

Table S3__________________________________________________________________________SI-4

Table S4__________________________________________________________________________SI-5

Table S5__________________________________________________________________________SI-7

Table S6__________________________________________________________________________SI-8

Table S7_________________________________________________________________________SI-10

Table S8_________________________________________________________________________SI-11

Table S9_________________________________________________________________________SI-14

Table S10________________________________________________________________________SI-15

Fig. S1__________________________________________________________________________SI-17

Fig. S2__________________________________________________________________________SI-18

Fig. S3__________________________________________________________________________SI-19

**Table S1: The average maximum dB of each focal species clip played during the experiment.** On the FoxPro, the volume settings were adjusted as follows: 14 for low playback volume, 27 for medium playback volume, and 40 for high playback volume, which is the maximum volume setting on the device.

| **Species Name** | **Species Code** | **Clip** | **Playback Level** | | |
| --- | --- | --- | --- | --- | --- |
|  |  |  | **Low** | **Medium** | **High** |
|  |  |  | **Average Max Decibels (dB)** | | |
| Ring-necked Pheasant | PHCO | A | 82.2 ± 0.6 | 107.1 ± 13.7 | 128.0 ± 3.5 |
|  |  | B | 62.4 ± 0.1 | 80.0 ± 0.2 | 113.3 ± 0.6 |
|  |  | C | 72.1 ± 0.3 | 91.0 ± 0.6 | 113.7 ± 1.1 |
| Northern Bobwhite | COVI | A | 79.7 ± 0.7 | 96.9 ± 13.2 | 118.0 ± 2.0 |
|  |  | B | 56.6 ± 0.2 | 75.6 ± 0.3 | 89.9 ± 0.0 |
|  |  | C | 74.0 ± 1.14 | 90.2 ± 2.1 | 116.3 ± 2.1 |
| Wild Turkey | MEGA | A | 67.8 ± 0.8 | 85.7 ± 0.9 | 116.0 ± 0.0 |
|  |  | B | 60.2 ± 0.2 | 80.6 ± 0.7 | 98.8 ± 12.3 |
|  |  | C | 60.1 ± 0.1 | 79.2 ± 0.5 | 113.0 ± 0.0 |
| Grasshopper Sparrow | AMSA | A | 51.3 ± 0.6 | 65.6 ± 0.5 | 82.5 ± 0.2 |
|  |  | B | 51.3 ± 0.2 | 60.2 ± 0.1 | 74.9 ± 0.1 |
|  |  | C | 52.0 ± 0.6 | 62.4 ± 0.0 | 76.8 ± 0.0 |
| Mourning Dove | ZEMA | A | 57.1 ± 0.1 | 77.2 ± 0.3 | 114.7 ± 1.5 |
|  |  | B | 68.3 ± 0.7 | 87.9 ± 0.3 | 122.0 ± 0.0 |
|  |  | C | 52.6 ± 0.1 | 70.4 ± 0.1 | 89.6 ± 0.8 |
| Dickcissel | SPAM | A | 62.0 ± 0.3 | 79.8 ± 0.3 | 89.9 ± 1.4 |
|  |  | B | 59.3 ± 0.1 | 79.4 ± 0.1 | 113.3 ± 0.6 |
|  |  | C | 61.4 ± 0.3 | 79.4 ± 0.5 | 105.7 ± 12.6 |
| **Average:** | | | 62.8 ± 9.4 | 80.5 ± 11.8 | 104.3 ± 16.3 |

**Table S2: For Ring-necked Pheasant, model 4 was the top-performing model according to the full model output.** Asterisks denote significance of variables in the model (* ≤ 0.05, ** ≤ 0.01, *** ≤ 0.001) and playback level is represented by dB.

| **Species Code** | **Model #** | **AICc** | **w_i_** | **Fixed Effects** | **Estimate** | **Std. Error** | **z value** | **p-value** |
| --- | --- | --- | --- | --- | --- | --- | --- | --- |
| PHCO | 4 | 738.6 | 0.87 | *Intercept* | 0.83 | 0.25 | 3.36 | <0.001*** |
|  |  |  |  | Distance | -2.26 | 0.26 | -8.54 | <0.001*** |
|  |  |  |  | ARU 1 | -0.58 | 0.21 | -2.70 | 0.007** |
|  |  |  |  | Low dB | -5.39 | 0.73 | -7.43 | <0.001*** |
|  |  |  |  | Medium dB | -1.89 | 0.27 | -6.98 | <0.001*** |
|  |  |  |  | Distance: ARU 1 | 0.99 | 0.28 | 3.59 | <0.001*** |
|  |  |  |  | Distance: Low dB | -2.43 | 0.77 | -3.17 | 0.001*** |
|  |  |  |  | Distance: Medium dB | -1.08 | 0.37 | -2.89 | 0.004** |
|  |  |  |  | **Random Effects** | **Variance** | | **Std. Dev** | |
|  |  |  |  | Wind Speed | 0.07 | | 0.27 | |
| PHCO | 6 | 742.4 | 0.13 | **Fixed Effects** | **Estimate** | **Std. Error** | **z value** | **p-value** |
|  |  |  |  | *Intercept* | 0.78 | 0.28 | 2.82 | 0.005** |
|  |  |  |  | Distance | -2.19 | 0.29 | -7.43 | <0.001*** |
|  |  |  |  | ARU 1 | -0.50 | 0.29 | -1.73 | 0.08 |
|  |  |  |  | Low dB | -5.31 | 0.76 | -7.01 | <0.001*** |
|  |  |  |  | Medium dB | -1.82 | 0.37 | -4.95 | <0.001*** |
|  |  |  |  | Distance: ARU 1 | 0.91 | 0.33 | 2.74 | 0.006** |
|  |  |  |  | Distance: Low dB | -2.51 | 0.80 | -3.15 | 0.002** |
|  |  |  |  | Distance: Medium dB | -1.09 | 0.38 | -2.88 | 0.004** |
|  |  |  |  | ARU 1: Low dB | -0.31 | 0.62 | -0.50 | 0.61 |
|  |  |  |  | ARU 1: Medium dB | -0.11 | 0.47 | -0.25 | 0.80 |
|  |  |  |  | **Random Effects** | **Variance** | | **Std. Dev** | |
|  |  |  |  | Wind Speed | 0.07 | | 0.27 | |
| PHCO | 2 | 773.4 | 0.00 | **Fixed Effects** | **Estimate** | **Std. Error** | **z value** | **p-value** |
|  |  |  |  | *Intercept* | 0.48 | 0.23 | 2.02 | 0.04* |
|  |  |  |  | Distance | -1.64 | 0.18 | -9.09 | <0.001*** |
|  |  |  |  | Low dB | -5.12 | 0.68 | -7.49 | <0.001*** |
|  |  |  |  | Medium dB | -1.82 | 0.26 | -6.92 | <0.001*** |
|  |  |  |  | Distance: Low dB | -2.39 | 0.72 | -3.32 | <0.001*** |
|  |  |  |  | Distance: Medium dB | -1.04 | 0.36 | -2.86 | 0.004** |
|  |  |  |  | **Random Effects** | **Variance** | | **Std. Dev** | |
|  |  |  |  | Wind Speed | 0.11 | | 0.33 | |
| PHCO | 3 | 1008.8 | 0.00 | **Fixed Effects** | **Estimate** | **Std. Error** | **z value** | **p-value** |
|  |  |  |  | *Intercept* | -0.72 | 0.16 | -4.45 | <0.001*** |
|  |  |  |  | Distance | -2.02 | 0.19 | -10.53 | <0.001*** |
|  |  |  |  | ARU 1 | -0.34 | 0.18 | -1.85 | 0.06 |
|  |  |  |  | Distance: ARU 1 | 0.77 | 0.25 | 3.11 | 0.002** |
|  |  |  |  | **Random Effects** | **Variance** | | **Std. Dev** | |
|  |  |  |  | Wind Speed | 0.73 | | 0.15 | |
| PHCO | 1 | 1025.6 | 0.00 | **Fixed Effects** | **Estimate** | **Std. Error** | **z value** | **p-value** |
|  |  |  |  | *Intercept* | -0.90 | 0.16 | -5.72 | <0.001*** |
|  |  |  |  | Distance | -1.59 | 0.12 | -13.29 | <0.001*** |
|  |  |  |  | **Random Effects** | **Variance** | | **Std. Dev** | |
|  |  |  |  | Wind Speed | 0.05 | | 0.22 | |
| PHCO | 5 | 1333.5 | 0.00 | **Fixed Effects** | **Estimate** | **Std. Error** | **z value** | **p-value** |
|  |  |  |  | *Intercept* | -0.52 | 0.10 | -5.33 | <0.001*** |
|  |  |  |  | **Random Effects** | **Variance** | | **Std. Dev** | |
|  |  |  |  | Wind Speed | 0.02 | | 0.13 | |

**Table S3: For Northern Bobwhite, model 4 was the top-performing model according to the full model output.** Asterisks denote significance of variables in the model (* ≤ 0.05, ** ≤ 0.01, *** ≤ 0.001) and playback level is represented by dB.

| **Species Code** | **Model #** | **AICc** | **w_i_** | **Fixed Effects** | **Estimate** | **Std. Error** | **z value** | **p-value** |
| --- | --- | --- | --- | --- | --- | --- | --- | --- |
| COVI | 4 | 859.1 | 0.87 | *Intercept* | 1.62 | 0.20 | 8.102 | <0.001*** |
|  |  |  |  | Distance | -1.73 | 0.20 | -8.441 | <0.001*** |
|  |  |  |  | ARU 1 | 0.73 | 0.18 | -4.06 | <0.001*** |
|  |  |  |  | Low dB | -3.64 | 0.35 | -10.46 | <0.001*** |
|  |  |  |  | Medium dB | -1.60 | 0.22 | -7.27 | <0.001*** |
|  |  |  |  | Distance: ARU 1 | 0.78 | 0.21 | 3.60 | <0.001*** |
|  |  |  |  | Distance: Low dB | -1.27 | 0.41 | -3.09 | 0.002** |
|  |  |  |  | Distance: Medium dB | 0.27 | 0.27 | -2.69 | 0.007** |
|  |  |  |  | **Random Effects** | **Variance** | | **Std. Dev** | |
|  |  |  |  | Wind Speed | 0.01 | | 0.11 | |
| COVI | 6 | 862.9 | 0.13 | **Fixed Effects** | **Estimate** | **Std. Error** | **z value** | **p-value** |
|  |  |  |  | *Intercept* | 1.55 | 0.26 | 5.99 | <0.001*** |
|  |  |  |  | Distance | -1.70 | 0.23 | -7.47 | <0.001*** |
|  |  |  |  | ARU 1 | -0.62 | 0.31 | -2.00 | 0.04* |
|  |  |  |  | Low dB | -3.57 | 0.43 | -8.25 | <0.001*** |
|  |  |  |  | Medium dB | -1.46 | 0.34 | -4.27 | <0.001*** |
|  |  |  |  | Distance: ARU 1 | 0.74 | 0.25 | 2.90 | 0.004** |
|  |  |  |  | Distance: Low dB | -1.28 | 0.42 | -3.07 | 0.002** |
|  |  |  |  | Distance: Medium dB | -0.76 | 0.28 | -2.72 | 0.006** |
|  |  |  |  | ARU 1: Low dB | -0.12 | 0.51 | -0.24 | 0.81 |
|  |  |  |  | ARU 1: Medium dB | -0.23 | 0.45 | -0.51 | 0.61 |
|  |  |  |  | **Random Effects** | **Variance** | | **Std. Dev** | |
|  |  |  |  | Wind Speed | 0.01 | | 0.11 | |
| COVI | 2 | 894.0 | 0.00 | **Fixed Effects** | **Estimate** | **Std. Error** | **z value** | **p-value** |
|  |  |  |  | *Intercept* | 1.18 | 0.19 | 6.17 | <0.001*** |
|  |  |  |  | Distance | -1.28 | 0.15 | -8.37 | <0.001*** |
|  |  |  |  | Low dB | -3.50 | 0.33 | -10.50 | <0.001*** |
|  |  |  |  | Medium dB | -1.55 | 0.22 | -7.17 | <0.001*** |
|  |  |  |  | Distance: Low dB | -1.25 | 0.39 | -3.19 | 0.001*** |
|  |  |  |  | Distance: Medium dB | -0.70 | 0.27 | -2.60 | 0.009** |
|  |  |  |  | **Random Effects** | **Variance** | | **Std. Dev** | |
|  |  |  |  | Wind Speed | 0.04 | | 0.20 | |
| COVI | 3 | 1096.9 | 0.00 | **Fixed Effects** | **Estimate** | **Std. Error** | **z value** | **p-value** |
|  |  |  |  | *Intercept* | 0.08 | 0.12 | 0.64 | 0.52 |
|  |  |  |  | Distance | -1.67 | 0.15 | -10.91 | <0.001*** |
|  |  |  |  | ARU 1 | -0.53 | 0.15 | -3.42 | <0.001*** |
|  |  |  |  | Distance: ARU 1 | 0.66 | 0.20 | 3.35 | <0.001*** |
|  |  |  |  | **Random Effects** | **Variance** | | **Std. Dev** | |
|  |  |  |  | Wind Speed | 0.70 | | 4.87 | |
| COVI | 1 | 1123.9 | 0.00 | **Fixed Effects** | **Estimate** | **Std. Error** | **z value** | **p-value** |
|  |  |  |  | *Intercept* | -0.21 | 0.11 | -1.87 | 0.06 |
|  |  |  |  | Distance | -1.30 | 0.09 | -13.66 | <0.001*** |
|  |  |  |  | **Random Effects** | **Variance** | | **Std. Dev** | |
|  |  |  |  | Wind Speed | 0.02 | | 0.14 | |
| COVI | 5 | 1398.3 | 0.00 | **Fixed Effects** | **Estimate** | **Std. Error** | **z value** | **p-value** |
|  |  |  |  | *Intercept* | -0.08 | 0.06 | -1.25 | 0.21 |
|  |  |  |  | **Random Effects** | **Variance** | | **Std. Dev** | |
|  |  |  |  | Wind Speed | 0.00 | | 0.03 | |

**Table S4: For Wild Turkey, model 4 was the top-performing model according to the full model output.** Asterisks denote significance of variables in the model (* ≤ 0.05, ** ≤ 0.01, *** ≤ 0.001) and playback level is represented by dB.

| **Species Code** | **Model #** | **AICc** | **w_i_** | **Fixed Effects** | **Estimate** | **Std. Error** | **z value** | **p-value** |
| --- | --- | --- | --- | --- | --- | --- | --- | --- |
| MEGA | 4 | 633.5 | 0.88 | *Intercept* | -0.19 | 0.31 | -0.64 | 0.52 |
|  |  |  |  | Distance | -3.11 | 0.37 | -8.49 | <0.001*** |
|  |  |  |  | ARU 1 | -0.28 | 0.29 | -0.97 | 0.33 |
|  |  |  |  | Low dB | -6.79 | 1.29 | -5.26 | <0.001*** |
|  |  |  |  | Medium dB | -1.95 | 0.36 | -5.43 | <0.001*** |
|  |  |  |  | Distance: ARU 1 | 1.16 | 0.38 | 3.01 | 0.003** |
|  |  |  |  | Distance: Low dB | -3.09 | 1.25 | -2.46 | 0.01** |
|  |  |  |  | Distance: Medium dB | -0.77 | 0.49 | -1.58 | 0.11 |
|  |  |  |  | **Random Effects** | **Variance** | | **Std. Dev** | |
|  |  |  |  | Wind Speed | 0.11 | | 0.34 | |
| MEGA | 6 | 637.5 | 0.12 | **Fixed Effects** | **Estimate** | **Std. Error** | **z value** | **p-value** |
|  |  |  |  | *Intercept* | -0.19 | 0.32 | -0.60 | 0.55 |
|  |  |  |  | Distance | -3.15 | 0.41 | -7.58 | <0.001*** |
|  |  |  |  | ARU 1 | -0.28 | 0.33 | -0.85 | 0.40 |
|  |  |  |  | Low dB | -6.81 | 1.29 | -5.27 | <0.001*** |
|  |  |  |  | Medium dB | -1.95 | 0.43 | -4.50 | <0.001*** |
|  |  |  |  | Distance: ARU 1 | 1.21 | 0.46 | 2.63 | 0.008** |
|  |  |  |  | Distance: Low dB | -3.04 | 1.26 | -2.41 | 0.02* |
|  |  |  |  | Distance: Medium dB | -0.77 | 0.49 | -1.55 | 0.12 |
|  |  |  |  | ARU 1: Low dB | 0.18 | 0.71 | 0.26 | 0.80 |
|  |  |  |  | ARU 1: Medium dB | -0.00 | 0.49 | -0.01 | 0.99 |
|  |  |  |  | **Random Effects** | **Variance** | | **Std. Dev** | |
|  |  |  |  | Wind Speed | 0.11 | | 0.34 | |
| MEGA | 2 | 658.4 | 0.00 | **Fixed Effects** | **Estimate** | **Std. Error** | **z value** | **p-value** |
|  |  |  |  | *Intercept* | -0.35 | 0.27 | -1.28 | 0.20 |
|  |  |  |  | Distance | -2.39 | 0.26 | -9.31 | <0.001*** |
|  |  |  |  | Low dB | -6.51 | 1.23 | -5.30 | <0.001*** |
|  |  |  |  | Medium dB | -1.87 | 0.35 | -5.39 | <0.001*** |
|  |  |  |  | Distance: Low dB | -3.09 | 1.19 | -2.59 | 0.009** |
|  |  |  |  | Distance: Medium dB | -0.74 | 0.47 | -1.56 | 0.12 |
|  |  |  |  | **Random Effects** | **Variance** | | **Std. Dev** | |
|  |  |  |  | Wind Speed | 0.14 | | 0.38 | |
| MEGA | 3 | 880.0 | 0.00 | **Fixed Effects** | **Estimate** | **Std. Error** | **z value** | **p-value** |
|  |  |  |  | *Intercept* | -1.57 | 0.21 | -7.31 | <0.001*** |
|  |  |  |  | Distance | -2.50 | 0.25 | -9.88 | <0.001*** |
|  |  |  |  | ARU 1 | -0.15 | 0.25 | -0.59 | 0.55 |
|  |  |  |  | Distance: ARU 1 | 0.79 | 0.33 | 2.39 | 0.02* |
|  |  |  |  | **Random Effects** | **Variance** | | **Std. Dev** | |
|  |  |  |  | Wind Speed | 0.03 | | 0.18 | |
| MEGA | 1 | 895.1 | 0.00 | **Fixed Effects** | **Estimate** | **Std. Error** | **z value** | **p-value** |
|  |  |  |  | *Intercept* | -1.63 | 0.19 | -8.74 | <0.001*** |
|  |  |  |  | Distance | -2.07 | 0.16 | -12.75 | <0.001*** |
|  |  |  |  | **Random Effects** | **Variance** | | **Std. Dev** | |
|  |  |  |  | Wind Speed | 0.06 | | 0.24 | |
| MEGA | 5 | 1224.5 | 0.00 | **Fixed Effects** | **Estimate** | **Std. Error** | **z value** | **p-value** |
|  |  |  |  | *Intercept* | -0.88 | 0.10 | -8.69 | <0.001*** |
|  |  |  |  | **Random Effects** | **Variance** | | **Std. Dev** | |
|  |  |  |  | Wind Speed | 0.02 | | 0.13 | |

**Table S5: For Dickcissel, models 4 and 6 were the top-performing models according to the full mode output.** Asterisks denote significance of variables in the model (* ≤ 0.05, ** ≤ 0.01, *** ≤ 0.001) and playback level is represented by dB.

| **Species Code** | **Model #** | **AICc** | **w_i_** | **Fixed Effects** | **Estimate** | **Std. Error** | **z value** | **p-value** |
| --- | --- | --- | --- | --- | --- | --- | --- | --- |
| SPAM | 4 | 680.4 | 0.68 | *Intercept* | 0.39 | 0.22 | 1.71 | 0.09 |
|  |  |  |  | Distance | -3.19 | 0.35 | -9.12 | <0.001*** |
|  |  |  |  | ARU 1 | -0.08 | 0.24 | -0.35 | 0.72 |
|  |  |  |  | Low dB | -5.62 | 0.80 | -7.05 | <0.001*** |
|  |  |  |  | Medium dB | -1.58 | 0.27 | -5.80 | <0.001*** |
|  |  |  |  | Distance: ARU 1 | 1.51 | 0.34 | 4.40 | <0.001*** |
|  |  |  |  | Distance: Low dB | -2.39 | 0.84 | -2.82 | 0.005** |
|  |  |  |  | Distance: Medium dB | -0.61 | 0.40 | -1.51 | 0.13 |
|  |  |  |  | **Random Effects** | **Variance** | | **Std. Dev** | |
|  |  |  |  | Wind Speed | 0.01 | | 0.09 | |
| SPAM | 6 | 681.9 | 0.31 | **Fixed Effects** | **Estimate** | **Std. Error** | **z value** | **p-value** |
|  |  |  |  | *Intercept* | 0.59 | 0.28 | 2.08 | 0.04 |
|  |  |  |  | Distance | -3.54 | 0.44 | -8.02 | <0.001*** |
|  |  |  |  | ARU 1 | -0.39 | 0.34 | -1.14 | 0.25 |
|  |  |  |  | Low dB | -6.08 | 0.84 | -7.20 | <0.001*** |
|  |  |  |  | Medium dB | -1.96 | 0.43 | -4.52 | <0.001*** |
|  |  |  |  | Distance: ARU 1 | 1.93 | 0.45 | 4.25 | <0.001*** |
|  |  |  |  | Distance: Low dB | -2.15 | 0.83 | -2.58 | 0.01** |
|  |  |  |  | Distance: Medium dB | -0.56 | 0.40 | -1.38 | 0.17 |
|  |  |  |  | ARU 1: Low dB | 1.04 | 0.68 | 1.54 | 0.12 |
|  |  |  |  | ARU 1: Medium dB | 0.57 | 0.51 | 1.11 | 0.26 |
|  |  |  |  | **Random Effects** | **Variance** | | **Std. Dev** | |
|  |  |  |  | Wind Speed | 0.01 | | 0.11 | |
| SPAM | 2 | 710.5 | 0.00 | **Fixed Effects** | **Estimate** | **Std. Error** | **z value** | **p-value** |
|  |  |  |  | *Intercept* | 0.33 | 0.18 | 1.80 | 0.07 |
|  |  |  |  | Distance | -2.24 | 0.24 | -9.42 | <0.001*** |
|  |  |  |  | Low dB | -5.40 | 0.75 | -7.19 | <0.001*** |
|  |  |  |  | Medium dB | -1.54 | 0.27 | -5.73 | <0.001*** |
|  |  |  |  | Distance: Low dB | -2.39 | 0.79 | -3.00 | 0.003** |
|  |  |  |  | Distance: Medium dB | -0.61 | 0.40 | -1.50 | 0.13 |
|  |  |  |  | **Random Effects** | **Variance** | | **Std. Dev** | |
|  |  |  |  | Wind Speed | 0.03 | | 0.18 | |
| SPAM | 3 | 936.3 | 0.00 | **Fixed Effects** | **Estimate** | **Std. Error** | **z value** | **p-value** |
|  |  |  |  | *Intercept* | -1.06 | 0.17 | -6.39 | <0.001*** |
|  |  |  |  | Distance | -2.62 | 0.24 | -10.71 | <0.001*** |
|  |  |  |  | ARU 1 | 0.02 | 0.21 | 0.12 | 0.90 |
|  |  |  |  | Distance: ARU 1 | 1.12 | 0.30 | 3.81 | <0.001*** |
|  |  |  |  | **Random Effects** | **Variance** | | **Std. Dev** | |
|  |  |  |  | Wind Speed | 0.00 | | 0.00 | |
| SPAM | 1 | 957.4 | 0.00 | **Fixed Effects** | **Estimate** | **Std. Error** | **z value** | **p-value** |
|  |  |  |  | *Intercept* | -1.02 | 0.12 | -9.27 | <0.001*** |
|  |  |  |  | Distance | -1.96 | 0.14 | -14.03 | <0.001*** |
|  |  |  |  | **Random Effects** | **Variance** | | **Std. Dev** | |
|  |  |  |  | Wind Speed | 0.00 | | 0.07 | |
| SPAM | 5 | 1340.8 | 0.00 | **Fixed Effects** | **Estimate** | **Std. Error** | **z value** | **p-value** |
|  |  |  |  | *Intercept* | -0.49 | 0.06 | -7.58 | <0.001*** |
|  |  |  |  | **Random Effects** | **Variance** | | **Std. Dev** | |
|  |  |  |  | Wind Speed | 0.00 | | 0.00 | |

**Table S6: For Grasshopper Sparrow, models 4 and 6 were the top performing models according to the full model output.** Asterisks denote significance of variables in the model (* ≤ 0.05, ** ≤ 0.01, *** ≤ 0.001) and playback level is represented by dB.

| **Species Code** | **Model #** | **AICc** | **w_i_** | **Fixed Effects** | **Estimate** | **Std. Error** | **z value** | **p-value** |
| --- | --- | --- | --- | --- | --- | --- | --- | --- |
| AMSA | 4 | 397.0 | 0.54 | *Intercept* | -2.39 | 0.50 | -4.78 | <0.001*** |
|  |  |  |  | Distance | -3.85 | 0.59 | -6.53 | <0.001*** |
|  |  |  |  | ARU 1 | -2.54 | 0.93 | -2.73 | 0.006** |
|  |  |  |  | Low dB | -8.74 | 4.02 | -2.17 | 0.03* |
|  |  |  |  | Medium dB | -2.73 | 1.01 | -2.70 | 0.007** |
|  |  |  |  | Distance: ARU 1 | -1.39 | 0.97 | -1.43 | 0.15 |
|  |  |  |  | Distance: Low dB | -4.55 | 3.58 | -1.27 | 0.20 |
|  |  |  |  | Distance: Medium dB | -0.80 | 1.05 | -0.76 | 0.45 |
|  |  |  |  | **Random Effects** | **Variance** | | **Std. Dev** | |
|  |  |  |  | Wind Speed | 0.11 | | 0.32 | |
| AMSA | 6 | 397.3 | 0.46 | **Fixed Effects** | **Estimate** | **Std. Error** | **z value** | **p-value** |
|  |  |  |  | *Intercept* | -2.26 | 0.48 | -4.67 | <0.001*** |
|  |  |  |  | Distance | -3.42 | 0.59 | -5.83 | <0.001*** |
|  |  |  |  | ARU 1 | -2.92 | 0.99 | -2.94 | 0.003** |
|  |  |  |  | Low dB | -9.19 | 4.12 | -2.23 | 0.02* |
|  |  |  |  | Medium dB | -2.75 | 1.01 | -2.72 | 0.006** |
|  |  |  |  | Distance: ARU 1 | -2.35 | 1.14 | -2.05 | 0.04* |
|  |  |  |  | Distance: Low dB | -5.52 | 3.70 | -1.49 | 0.13 |
|  |  |  |  | Distance: Medium dB | -1.22 | 1.10 | -1.10 | 0.27 |
|  |  |  |  | ARU 1: Low dB | -1.97 | 1.25 | -1.58 | 0.11 |
|  |  |  |  | ARU 1: Medium dB | -0.92 | 0.68 | -1.34 | 0.18 |
|  |  |  |  | **Random Effects** | **Variance** | | **Std. Dev** | |
|  |  |  |  | Wind Speed | 0.12 | | 0.35 | |
| AMSA | 2 | 419.2 | 0.00 | **Fixed Effects** | **Estimate** | **Std. Error** | **z value** | **p-value** |
|  |  |  |  | *Intercept* | -3.15 | 0.48 | -6.57 | <0.001*** |
|  |  |  |  | Distance | -3.97 | 0.52 | -7.62 | <0.001*** |
|  |  |  |  | Low dB | -8.55 | 3.98 | -2.14 | 0.03* |
|  |  |  |  | Medium dB | -2.64 | 1.00 | -2.64 | 0.008** |
|  |  |  |  | Distance: Low dB | -4.56 | 3.55 | -1.29 | 0.20 |
|  |  |  |  | Distance: Medium dB | -0.83 | 1.04 | -0.80 | 0.42 |
|  |  |  |  | **Random Effects** | **Variance** | | **Std. Dev** | |
|  |  |  |  | Wind Speed | 0.16 | | 0.40 | |
| AMSA | 3 | 529.2 | 0.00 | **Fixed Effects** | **Estimate** | **Std. Error** | **z value** | **p-value** |
|  |  |  |  | *Intercept* | -3.40 | 0.39 | -8.79 | <0.001*** |
|  |  |  |  | Distance | -3.13 | 0.41 | -7.58 | <0.001*** |
|  |  |  |  | ARU 1 | -2.16 | 0.83 | -2.60 | 0.009** |
|  |  |  |  | Distance: ARU 1 | -1.31 | 0.85 | -1.54 | 0.12 |
|  |  |  |  | **Random Effects** | **Variance** | | **Std. Dev** | |
|  |  |  |  | Wind Speed | 0.03 | | 0.19 | |
| AMSA | 1 | 545.9 | 0.00 | **Fixed Effects** | **Estimate** | **Std. Error** | **z value** | **p-value** |
|  |  |  |  | *Intercept* | -4.07 | 0.37 | -11.02 | <0.001*** |
|  |  |  |  | Distance | -3.41 | 0.35 | -9.64 | <0.001*** |
|  |  |  |  | **Random Effects** | **Variance** | | **Std. Dev** | |
|  |  |  |  | Wind Speed | 0.08 | | 0.28 | |
| AMSA | 5 | 785.3 | 0.00 | **Fixed Effects** | **Estimate** | **Std. Error** | **z value** | **p-value** |
|  |  |  |  | *Intercept* | -1.91 | 0.14 | -13.78 | <0.001*** |
|  |  |  |  | **Random Effects** | **Variance** | | **Std. Dev** | |
|  |  |  |  | Wind Speed | 0.03 | | 0.17 | |

**Table S7: For Mourning Dove, models 4 and 6 were the top-performing models according to the full model output.** Asterisks denote significance of variables in the model (* ≤ 0.05, ** ≤ 0.01, *** ≤ 0.001) and playback level is represented by dB. Low dB was not included in these models.

| **Species Code** | **Model #** | **AICc** | **w_i_** | **Fixed Effects** | **Estimate** | **Std. Error** | **z value** | **p-value** |
| --- | --- | --- | --- | --- | --- | --- | --- | --- |
| ZEMA | 4 | 349.4 | 0.67 | *Intercept* | -2.44 | 0.51 | -4.82 | <0.001*** |
|  |  |  |  | Distance | -4.47 | 0.67 | -6.62 | <0.001*** |
|  |  |  |  | ARU 1 | -0.86 | 0.76 | -1.14 | 0.25 |
|  |  |  |  | Medium dB | -3.97 | 1.26 | -3.15 | 0.001** |
|  |  |  |  | Distance: ARU 1 | 1.13 | 0.88 | 1.29 | 0.19 |
|  |  |  |  | Distance: Medium dB | -1.41 | 1.28 | -1.10 | 0.27 |
|  |  |  |  | **Random Effects** | **Variance** | | **Std. Dev** | |
|  |  |  |  | Wind Speed | 0.04 | | 0.20 | |
| ZEMA | 6 | 351.0 | 0.33 | **Fixed Effects** | **Estimate** | **Std. Error** | **z value** | **p-value** |
|  |  |  |  | *Intercept* | -2.36 | 0.50 | -4.70 | <0.001*** |
|  |  |  |  | Distance | -4.23 | 0.71 | -5.95 | <0.001*** |
|  |  |  |  | ARU 1 | -0.96 | 0.77 | -1.25 | 0.21 |
|  |  |  |  | Medium dB | -4.00 | 1.29 | -3.11 | 0.001*** |
|  |  |  |  | Distance: ARU 1 | 0.80 | 0.98 | 0.82 | 0.41 |
|  |  |  |  | Distance: Medium dB | -1.69 | 1.36 | -1.24 | 0.21 |
|  |  |  |  | ARU 1: Medium dB | -0.61 | 0.79 | -0.76 | 0.44 |
|  |  |  |  | **Random Effects** | **Variance** | | **Std. Dev** | |
|  |  |  |  | Wind Speed | 0.05 | | 0.21 | |
| ZEMA | 2 | 383.0 | 0.00 | **Fixed Effects** | **Estimate** | **Std. Error** | **z value** | **p-value** |
|  |  |  |  | *Intercept* | -2.66 | 0.42 | -6.36 | <0.001*** |
|  |  |  |  | Distance | -3.53 | 0.46 | -7.73 | <0.001*** |
|  |  |  |  | Medium dB | -3.78 | 1.18 | -3.21 | 0.001*** |
|  |  |  |  | Distance: Medium dB | -1.5 | 1.17 | -1.31 | 0.19 |
|  |  |  |  | **Random Effects** | **Variance** | | **Std. Dev** | |
|  |  |  |  | Wind Speed | 0.14 | | 0.37 | |
| ZEMA | 3 | 538.8 | 0.00 | **Fixed Effects** | **Estimate** | **Std. Error** | **z value** | **p-value** |
|  |  |  |  | *Intercept* | -3.09 | 0.41 | -7.49 | <0.001*** |
|  |  |  |  | Distance | -3.71 | 0.50 | -7.42 | <0.001*** |
|  |  |  |  | ARU 1 | -0.82 | 0.69 | -1.18 | 0.24 |
|  |  |  |  | Distance: ARU 1 | 0.73 | 0.78 | 0.94 | 0.35 |
|  |  |  |  | **Random Effects** | **Variance** | | **Std. Dev** | |
|  |  |  |  | Wind Speed | 0 | | 0 | |
| ZEMA | 1 | 560.1 | 0.00 | **Fixed Effects** | **Estimate** | **Std. Error** | **z value** | **p-value** |
|  |  |  |  | *Intercept* | -3.33 | 0.34 | -9.79 | <0.001*** |
|  |  |  |  | Distance | -3.17 | 0.35 | -8.99 | <0.001*** |
|  |  |  |  | **Random Effects** | **Variance** | | **Std. Dev** | |
|  |  |  |  | Wind Speed | 0.05 | | 0.23 | |
| ZEMA | 5 | 755.0 | 0.00 | **Fixed Effects** | **Estimate** | **Std. Error** | **z value** | **p-value** |
|  |  |  |  | *Intercept* | -1.52 | 0.16 | -9.56 | <0.001*** |
|  |  |  |  | **Random Effects** | **Variance** | | **Std. Dev** | |
|  |  |  |  | Wind Speed | 0.04 | | 0.21 | |

**Table S8: Detection probability for each ARU and focal species across playback levels.** Asterisks denote significance between ARUs (* ≤ 0.05, ** ≤ 0.01, *** ≤ 0.001).

| Species Code | Playback level | Percent Probability | | p-value |
| --- | --- | --- | --- | --- |
|  |  | *ARU #1* | *ARU #2* |  |
| PHCO | Low | 0.58 ± 0.00 | 1.03 ± 0.01 | <0.001*** |
|  | Medium | 16.20 ± 0.04 | 25.66 ± 0.06 | 0.06 |
|  | High | 56.21 ± 0.06 | 69.63 ± 0.05 | <0.001*** |
| MEGA | Low | 0.07 ± 0.00 | 0.09 ± 0.00 | <0.001*** |
|  | Medium | 8.15 ± 0.03 | 10.50 ± 0.04 | 0.04 |
|  | High | 38.38 ± 0.07 | 45.14 ± 0.08 | <0.001*** |
| COVI | Low | 5.97 ± 0.02 | 11.67 ± 0.03 | <0.001*** |
|  | Medium | 33.04 ± 0.04 | 50.68 ± 0.05 | 0.33 |
|  | High | 70.90 ± 0.04 | 83.54 ± 0.03 | <0.001*** |
| SPAM | Low | 0.49 ± 0.00 | 0.53 ± 0.00 | <0.001*** |
|  | Medium | 21.73 ± 0.04 | 23.23 ± 0.05 | 0.01* |
|  | High | 57.46 ± 0.05 | 59.54 ± 0.05 | <0.001*** |
| AMSA | Low | 0.00 ± 0.00 | 0.00 ± 0.00 | 0.10 |
|  | Medium | 0.05 ± 0.00 | 0.60 ± 0.01 | 0.46 |
|  | High | 0.72 ± 0.01 | 8.42 ± 0.04 | 0.02 |
| ZEMA | Low | NA | NA | NA |
|  | Medium | 0.07 ± 0.00 | 0.16 ± 0.00 | 0.002** |
|  | High | 3.53 ± 0.02 | 8.00 ± 0.04 | 0.002** |

**Table S9: Probability of detection for each direction and focal species.**

| **Species Code** | **Direction** | **Detection Probability** |
| --- | --- | --- |
| PHCO | E | 0.37 ± 0.04 |
|  | N | 0.45 ± 0.04 |
|  | S | 0.49 ± 0.04 |
|  | W | 0.31 ± 0.03 |
| MEGA | E | 0.26 ± 0.03 |
|  | N | 0.28 ± 0.03 |
|  | S | 0.39 ± 0.04 |
|  | W | 0.19 ± 0.03 |
| COVI | E | 0.53 ± 0.04 |
|  | N | 0.56 ± 0.03 |
|  | S | 0.62 ± 0.03 |
|  | W | 0.49 ± 0.04 |
| SPAM | E | 0.37 ± 0.03 |
|  | N | 0.46 ± 0.03 |
|  | S | 0.53 ± 0.04 |
|  | W | 0.27 ± 0.03 |
| AMSA | E | 0.04 ± 0.01 |
|  | N | 0.05 ± 0.01 |
|  | S | 0.06 ± 0.02 |
|  | W | 0.05 ± 0.01 |
| ZEMA | E | 0.03 ± 0.01 |
|  | N | 0.06 ± 0.01 |
|  | S | 0.08 ± 0.02 |
|  | W | 0.07 ± 0.02 |

**Table S10: Significant differences in detection probability between directions for each focal species.** Asterisks denote significance between ARUs (* ≤ 0.05, ** ≤ 0.01, *** ≤ 0.001).

| **Species Code** | **Contrast** | **p-value** |
| --- | --- | --- |
| PHCO | E/N | 0.45 |
|  | E/S | 0.14 |
|  | E/W | 0.49 |
|  | N/S | 0.90 |
|  | N/W | 0.02* |
|  | S/W | <0.01** |
| MEGA | E/N | 0.97 |
|  | E/S | 0.03* |
|  | E/W | 0.34 |
|  | N/S | 0.09 |
|  | N/W | 0.15 |
|  | S/W | <0.001*** |
| COVI | E/N | 0.93 |
|  | E/S | 0.30 |
|  | E/W | 0.89 |
|  | N/S | 0.65 |
|  | N/W | 0.55 |
|  | S/W | 0.07 |
| SPAM | E/N | 0.34 |
|  | E/S | 0.01* |
|  | E/W | 0.17 |
|  | N/S | 0.58 |
|  | N/W | 0.001*** |
|  | S/W | <0.001*** |
| AMSA | E/N | 0.83 |
|  | E/S | 0.27 |
|  | E/W | 0.70 |
|  | N/S | 0.77 |
|  | N/W | 0.99 |
|  | S/W | 0.88 |
| ZEMA | E/N | 0.19 |
|  | E/S | 0.02* |
|  | E/W | 0.04* |
|  | N/S | 0.77 |
|  | N/W | 0.91 |
|  | S/W | 0.99 |

**Fig. S1: ARU #1 in Management Parcel #1.**

**Fig. S2: ARU #2 in Management Parcel #2.**

**Fig. S3: Predictions based on the top-performing model(s) for each focal species across ARUs, without incorporating variation in playback level.** The red dashed line represents average detection probability across ARU for each species. ‘Distance’ represents the distance between the monitor and the source of the vocalization.
